# Active commuting and perceptions of the route environment: A longitudinal analysis

**DOI:** 10.1016/j.ypmed.2014.06.033

**Published:** 2014-10

**Authors:** Jenna Panter, Simon Griffin, David Ogilvie

**Affiliations:** Medical Research Council Epidemiology Unit and UKCRC, Centre for Diet and Activity Research (CEDAR), University of Cambridge School of Clinical Medicine, Box 285, Cambridge Biomedical Campus, Cambridge CB2 0QQ, UK

**Keywords:** Environment design, Health promotion, Physical activity, Walking

## Abstract

**Objective:**

To assess associations between changes in perceptions of the environment en route to work and changes in active commuting.

**Methods:**

655 commuters in Cambridge, UK reported perceptions of their commuting route and past-week commuting trips in postal questionnaires in 2009 and 2010. Associations between changes in route perceptions and changes in time spent walking and cycling, proportion of car trips, and switching to or from the car on the commute were modelled using multivariable regression.

**Results:**

Changes in only a few perceptions were associated with changes in travel behaviour. Commuters who reported that it became less pleasant to walk recorded a 6% (95% CI: 1, 11) net increase in car trips and a 12 min/week (95% CI: − 1, − 24) net decrease in walking. Increases in the perceived danger of cycling or of crossing the road were also associated with increases in car trips. Increases in the perceived convenience of public transport (OR: 3.31, 95% CI: 1.27, 8.63) or safety of cycling (OR: 3.70, 95% CI: 1.44, 9.50) were associated with taking up alternatives to the car.

**Conclusions:**

Interventions to improve the safety of routes and convenience of public transport may help promote active commuting and should be evaluated.

## Highlights

• Changes in certain perceptions of routes were associated with changes in commuting.
• Reduced pleasantness for walking was associated with a decrease in walking.
• Increased convenience of public transport was associated with decreased car use.
• Increased danger for pedestrians and cyclists was associated with increased car use.
• Interventions to address these factors may help promote active commuting.

## Introduction

Promoting physical activity, including incidental activity incurred through transport, is a public health priority (Department of Health, 2011, US Department of Health and Services, 1996). However, evidence to support interventions to promote population shifts in travel behaviour is limited (Ogilvie et al., 2007, Yang et al., 2010). In a previous paper, we described how the longitudinal analysis of observational datasets could contribute to our understanding in this area, and demonstrated the importance of individual, household and environmental factors measured at baseline in predicting the uptake and maintenance of walking and cycling to work (Panter et al., 2013a). In this paper, we investigate a more specific association between changes in perceptions of the environment en route to work and changes in commuting behaviour.

One feature of the ecological model of health behaviour is the notion that the context in which behaviour is undertaken is important (Sallis and Owen, 2002). However, the mechanisms by which the environment influences behaviour change are poorly understood (Kremers et al., 2006): they may involve direct, unmediated processes, or be mediated by the cognitive processing and storage of environmental conditions (Kaplan and Kaplan, 1982). Perceptions of the environment represent one of the most proximal cognitive constructs that may change as a function of changing environments. Few studies have examined whether changes in environmental perceptions are associated with changes in physical activity; one found that university employees who reported improvements in the convenience of routes (and, among men, in their aesthetics) increased their walking (Humpel et al., 2004).

Changes in environmental perceptions may be reported in the presence or absence of an intervention. Understanding their relationship with behaviour change in observational studies can complement analyses of baseline predictors of change (Panter et al., 2013a) and, ultimately, intervention studies in elucidating the casual mechanisms linking environmental change to behaviour change (Bauman et al., 2002, McCormack and Shiell, 2011, Ogilvie et al., 2011). Greater understanding about which specific environmental attributes (and changes therein) are associated with behaviour change is crucial for informing the design and targeting of future interventions. It will also provide greater confidence in the significance and role of specific factors along the putative casual pathway for interventions (Pawson and Tiley, 1997). In this paper, we assess the associations between changes in perceptions of the environment en route to work and changes in walking, cycling and car use for commuting in a sample of working adults.

## Methods

### Study design

The recruitment and data collection procedures used in the *Commuting and Health in Cambridge* study have been described in detail (Ogilvie et al., 2010, Panter et al., 2011, Yang et al., 2012) and the entire questionnaire published elsewhere (Panter et al., 2011). Briefly, adults over the age of 16 working in Cambridge and living in urban or rural areas within 30 km of the city were recruited, predominantly through workplaces. Postal surveys were sent in May–October 2009 (t_1_) and again one year later (t_2_), matched to the same week wherever possible.

### Outcomes

At both time points participants were asked to report the travel modes used on each journey to and from work over the last seven days. If participants walked or cycled for any part of these journeys, they were asked to report the average time spent doing so per trip. We used this information to derive two suites of outcome variables:

#### Change in time spent walking or cycling or proportion of car trips

The total weekly times spent walking and cycling to and from work at t_1_ and t_2_ were computed (average duration ∗ number of trips), change scores (t_2_ − t_1_) were computed and those >±300 min/week were truncated to 300. The number of trips made using only the car at each time point was also computed and used to derive the relative change in the percentage of car-only trips ((t_2_ − t_1_) / t_1_).

#### Uptake of walking, cycling and alternatives to the car

Participants who reported an increase in time spent walking or cycling from zero at t_1_ were classified as having ‘taken up’ walking or cycling. The most frequently reported travel mode or combination of modes (referred to as ‘usual mode(s)’) used at each time point was also computed and used to identify participants who had shifted from usually using the car (at t_1_) to an alternative mode (at t_2_) (additional file A).

### Exposures: changes in perceptions of the route environment

A range of characteristics of the route to work were chosen because they represented constructs that were believed to be important determinants of behaviour (Panter and Jones, 2010, Pikora et al., 2003). Participants reported their level of agreement with seven statements describing the route environment using a five-point Likert scale at both t_1_ and t_2_ and the change in agreement for each item (t_2_ − t_1_) was computed.

### Covariates

Dates of birth and of questionnaire completion, gender, highest educational qualification, housing tenure, household composition, access to cars and bicycles, possession of a driving licence, limiting long term illness, height and weight were assessed by questionnaire. Age and season of data collection were calculated using the date of questionnaire completion and season was defined as either early summer (May–June), mid-summer (July–August) or autumn (September–October). Participants also reported their home and work postcodes, workplace car parking provision at both time points, and the occurrence of any life events (such as changes in household composition or work responsibilities) in the last year at t_2_. Responses were used to derive three binary variables indicating a change in workplace parking, a change in home or work location and the occurrence of any (other) life events.

### Analyses

We used t-tests to compare average perceptions between t_1_ and t_2_; a weighted kappa score (Sim and Wright, 2005) and percentage agreement (Chinn and Burney, 1987) to assess the within-participant agreement between t_1_ and t_2_ perception scores; and one-way analysis of variance (ANOVA) to assess the association between changes in perceptions and their baseline values. In all descriptive analyses we investigated differences by gender.

Separate linear regression models were used to assess the independent associations between changes in each of the route perceptions and changes in time spent walking, cycling and the proportion of car-only trips, initially minimally adjusted for age, gender, season and baseline travel behaviour. Given the uncertainty about the magnitude of environmental change required for behaviour change, participants were assigned to one of three groups: those who reported a less supportive condition at t_2_, those who reported a more supportive condition at t_2_; and those who reported no change. At this stage we also tested for interactions between environmental perceptions and gender. Although adjustment for baseline values of the outcome in analyses of change is subject to some debate (Fitzmaurice, 2001), our results were consistent in terms of effect size and statistical significance with and without adjustment. All variables associated at *p* < 0.25 (Hosmer and Lemeshow, 1989) were carried forward into multivariable regression models, additionally adjusted for changes in workplace car parking and home or work location and other life events. As a sensitivity analysis, we also examined whether these adjusted associations varied by the magnitude of perceived change. We used three logistic regression models to explore whether changes in perceptions were associated with uptake of walking, cycling and use of alternatives to the car, following the same approach to model building. Interactions were not fitted in logistic regression models because of small sample sizes, and *p*-values were not adjusted for (limited) multiple testing in the final multivariable models because this was intended as an exploratory analysis of plausible associations rather than a conclusive analysis of ‘effects’ and the practice is subject to debate (Feise, 2002).

## Results

### Sample characteristics

Of the 1142 participants who provided information on commuting at t_1_, 655 did so again at t_2_ and were included in this analysis. Those providing data at follow-up were more likely to be older and to own their home than those who did not, but there were no other significant differences in socioeconomic characteristics or baseline levels of active commuting (Panter et al., 2013a). Participants were aged between 17 and 70 years at t_1_ (mean age 43.6 years, s.d 11.3), 69% were women and 74% reported having at least degree-level education. Further details of the characteristics of the sample and their travel are given in additional file B and elsewhere (Panter et al., 2013a).

### Changes in environmental perceptions

The only significant change in mean perception scores over time was that women (but not men) reported that it was less pleasant to walk at t_2_ than at t_1_ (Table 1). The mean within-participant change scores were also small. Within-participant agreement between perceptions reported at t_1_ and t_2_ was moderate (based on weighted kappa scores) (Landis and Koch, 1977) or fair (based on percentage agreement) (Table 2) (Portney and Watkins, 2000). Participants who reported less favourable perceptions at t_1_ tended to report greater increases in perception scores, whereas those with initially more positive perceptions tended to report stable or decreasing scores (Table 3).

**Table 1:** Baseline and follow-up scores for perceptions of the route environment.

Perception of the route environment	All participants			Men			Women			Within-participant change Mean (SD)
	t_1_ Mean (SD)	t_2_ Mean (SD)	*p*^a^	t_1_ Mean (SD)	t_2_ Mean (SD)	*p*^b^	t_1_ Mean (SD)	t_2_ Mean (SD)	*p*^c^	
It is pleasant to walk	3.47 (1.19)	3.36 (1.23)	0.010	3.46 (1.20)	3.40 (1.23)	0.222	3.47 (1.18)	3.35 (3.23)	0.025	0.11 (1.12)
The roads are dangerous for cyclists*	2.50 (1.09)	2.57 (1.13)	0.159	2.68 (1.11)	2.71 (1.20)	0.793	2.42 (1.06)	2.50 (1.09)	0.127	0.06 (1.07)
There is convenient public transport	2.74 (1.29)	2.68 (1.29)	0.210	2.55 (1.28)	2.55 (1.26)	0.946	2.82 (1.28)	2.73 (1.29)	0.123	0.05 (1.07)
There are convenient cycle routes	3.31 (1.21)	3.30 (1.22)	0.943	3.35 (1.15)	3.37 (1.17)	0.781	3.28 (1.24)	3.26 (1.24)	0.933	− 0.01 (1.10)
There are no convenient routes for walking*	3.33 (1.25)	3.39 (1.24)	0.213	3.35 (1.20)	3.40 (1.24)	0.696	3.32 (1.27)	3.39 (1.25)	0.220	0.06 (1.30)
There is little traffic	1.84 (1.03)	1.84 (1.03)	0.969	1.98 (1.08)	1.97 (1.09)	0.829	1.76 (0.99)	1.77 (0.99)	0.928	0.01 (1.03)
It is safe to cross the road	3.25 (1.04)	3.29 (1.07)	0.410	3.34 (0.95)	3.43 (0.98)	0.241	3.21 (1.07)	3.22 (1.10)	0.774	− 0.04 (1.10)

These items were specifically related to the route to work, such that each was prefaced in the questionnaire with the stem ‘On my journey to and from work…’ For all items, scores are coded such that higher values indicate more supportive environments (1: strongly disagree, 2: disagree, 3: neither agree nor disagree, 4: agree, 5: strongly agree, except for * where the coding is reversed). Data collected in 2009 and 2010 in Cambridge, UK.

a Difference between t_1_ and t_2_.

b Difference between t_1_ and t_2_ for men.

c Difference between t_1_ and t_2_ for women.

**Table 2:** Agreement between baseline and follow-up scores for perceptions of the route environment.

Perception of the route environment	All participants		Men		Women	
	% agreement	Kappa	% agreement	Kappa	% agreement	Kappa
It is pleasant to walk	82.4	0.46	80.3	0.41	83.2	0.49
The roads are dangerous for cyclists	82.5	0.42	82.5	0.45	82.5	0.41
There is convenient public transport	83.4	0.54	84.4	0.56	83.0	0.53
There are convenient cycle routes	83.1	0.49	84.9	0.52	82.3	0.48
There are no convenient routes for walking	79.1	0.38	79.1	0.38	79.1	0.39
There is little traffic	85.5	0.43	86.5	0.51	85.0	0.38
It is safe to cross the road	82.6	0.38	85.7	0.44	81.1	0.36

All items measured using a five-point Likert scale at each time point. Standard errors for all kappa scores ranged between 0.02 and 0.05. Data collected in 2009 and 2010 in Cambridge, UK.

**Table 3:** Mean changes in perceptions of the route environment stratified by baseline perceptions.

Perception of the route environment	All participants		Men		Women	
	Mean change (SD)	N	Mean change (SD)	N	Mean change (SD)	n
Pleasant to walk						
Reported unsupportive condition at t_1_ (D/SD)	0.73 (1.14)	126	0.74 (1.12)	42	0.72 (1.15)	84
Reported neutral condition at t_1_ (N)	0.12 (0.92)	157	0.24 (1.04)	45	0.07 (0.87)	112
Reported supportive condition at t_1_ (A/SA)	0.53 (0.99)	342	− 0.58 (1.00)	106	− 0.51 (0.97)	236
Convenient public transport						
Reported unsupportive condition at t_1_ (D/SD)	0.38 (1.01)	318	0.39 (1.03)	108	0.37 (1.01)	210
Reported neutral condition at t_1_ (N)	− 0.32 (0.97)	85	− 0.39 (0.90)	33	− 0.27 (1.03)	52
Reported supportive condition at t_1_ (A/SA)	− 0.54 (0.92)	237	− 0.47 (0.85)	59	− 0.56 (0.94)	178
Little traffic						
Reported unsupportive condition at t_1_ (D/SD)	0.19 (0.84)	534	0.15 (0.80)	161	0.21 (0.85)	373
Reported neutral condition at t_1_ (N)	− 0.43 (1.07)	55	− 0.22 (0.99)	23	− 0.59 (1.10)	32
Reported supportive condition at t_1_ (A/SA)	− 1.35 (1.38)	60	− 1.04 (1.43)	21	− 1.51 (1.35)	39
Convenient walking routes						
Reported unsupportive condition at t_1_ (A/SA)	0.99 (1.42)	161	0.92 (1.28)	49	1.02 (1.49)	112
Reported neutral condition at t_1_ (N)	0.36 (0.91)	136	0.31 (0.98)	42	0.38 (0.88)	94
Reported supportive condition at t_1_ (D/SD)	− 0.47 (1.06)	345	− 0.45 (1.08)	112	− 0.50 (1.05)	233
Safe to cross the road						
Reported unsupportive condition at t_1_ (D/SD)	0.85 (1.24)	149	0.86 (1.22)	37	0.84 (1.26)	112
Reported neutral condition (N)	0.18 (0.80)	180	0.22 (0.73)	62	0.17 (0.84)	118
Reported supportive condition at t_1_ (A/SA)	− 0.44 (0.91)	315	− 0.29 (0.76)	104	− 0.51 (0.97)	211
Convenient cycle routes						
Reported unsupportive condition at t_1_ (D/SD)	0.65 (1.12)	185	0.59 (1.06)	59	0.68 (1.14)	126
Reported neutral condition at t_1_ (N)	0.21 (0.90)	108	0.12 (0.82)	33	0.25 (0.93)	75
Reported supportive condition at t_1_ (A/SA)	− 0.40 (0.96)	352	− 0.31 (0.87)	113	− 0.44 (0.99)	239
Dangerous to cycle						
Reported unsupportive condition at t_1_ (A/SA)	0.41 (0.93)	374	0.44 (0.95)	104	0.40 (0.92)	270
Reported neutral condition at t_1_ (N)	− 0.18 (0.94)	117	− 0.35 (0.98)	39	− 0.09 (0.91)	78
Reported supportive condition at t_1_ (D/SD)	− 0.64 (1.09)	149	− 0.46 (1.03)	60	− 0.76 (1.11)	89

D/SD: disagree or strongly disagree; N: neither; A/SA: agree or strongly agree. The mean change values differed significantly according to condition reported at baseline (p < 0.004 for all items) for all participants, as well as for men and women separately. Data collected in 2009 and 2010 in Cambridge, UK.

### Associations between changes in environmental perceptions and changes in commuting behaviour

#### Change in time spent walking or cycling or proportion of car trips

Minimally-adjusted regression models suggested that changes in only a few perceptions of the route environment were associated with changes in commuting (Table 4). The unadjusted means illustrate the average changes in time spent walking and cycling and in the proportion of car-only trips for each category of change in perceptions. Of all the interactions tested, only one was significant: an increase in convenience of walking routes over time was associated with a decrease in car trips in women (p = 0.02) but not men (p = 0.18). In maximally-adjusted models, reporting less pleasant walking routes over time was associated with a net decrease in walking of 12 min/week (95% CI: − 1 to − 24) compared with those reporting no change. Reports that routes became less pleasant for walking or more dangerous for cycling, or became more dangerous to cross the road, were all associated with estimated relative increases in the proportion of car-only trips of 6% (95% CI 1 to 11), 8% (2 to 13) and 5% (0 to 10) respectively compared with those reporting no change. The associations observed for the magnitude of the change in perceptions (additional file C) were generally similar to those presented in Table 4.

**Table 4:** Associations between changes in route perceptions and changes in time spent walking and cycling and proportion of car-only trips.

	N	Change in time spent walking on the commute (min/week)			Change in time spent cycling on the commute (min/week)			Change in percentage of car-only trips		
			Model 1	Model 2		Model 1	Model 2		Model 1	Model 2
		Mean Δ	β coefficient (95% CI) *p*	β coefficient (95% CI) *p*	Mean Δ	β coefficient (95% CI) *p*	β coefficient (95% CI) *p*	Mean Δ	β coefficient (95% CI) *p*	β coefficient (95% CI) *p*
Pleasant to walk										
Less pleasant	181 (29.0)	− 5.72	− 11.23 (− 22.17, − 0.29)⁎	− 11.94 (− 23.49, − 0.63)⁎				5.93	5.90 (1.34, 10.50)⁎⁎	6.23 (1.27, 11.19)⁎⁎⁎
No change (reference)	312 (49.9)	5.81	0	0		NT	NT	− 1.31	0	0
More pleasant	132 (21.1)	10.57	1.32 (− 11.44, 14.08)	− 0.64 (− 13.43, 12.16)				0.69	2.67 (− 2.65, 7.99)	2.34 (− 3.13, 7.82)
Convenient public transport										
Less convenient public transport	168 (26.3)	3.88	3.58 (− 8.49, 15.64)	3.23 (− 8.51, 14.97)			3.50		1.59 (− 3.13, 6.31)	0.02 (− 5.03, 5.06)
No change (reference)	340 (53.1)	− 0.37	0	0		NT	1.22		0	0
More convenient public transport	132 (20.6)	11.48	11.76 (− 1.72, 25.23)†	10.52 (− 2.04, 23.09)†			− 1.94		− 3.26 (− 8.51, 2.00)†	− 4.06 (− 9.46, 1.34)†
Little traffic										
More traffic	147 (22.9)	6.58	0.78 (− 11.36, 12.91)		4.62	1.28 (− 4.15, 6.71)		1.25	− 0.11 (− 5.20, 4.98)	
No change (reference)	339 (52.8)	2.78	0	NS	− 7.27	0	NS	0.81	0	NS
Less traffic	156 (24.3)	0.13	− 1.71 (− 14.09, 10.67)		− 8.79	0.44 (− 4.95, 5.83)		2.09	0.04 (− 5.16, 5.24)	
Convenient walking routes										
Less convenient routes	163 (25.4)	7.86	9.08 (− 2.16, 20.32)†	6.64 (− 5.30, 18.57)				3.74	3.58 (− 1.34, 8.51)†	3.05 (− 1.99, 8.09)†
No change (reference)	295 (46.0)	− 3.03	0	0		NT	NT	1.08	0	0
More convenient routes	184 (28.6)	9.55	8.61 (− 3.33, 20.54)†	11.26 (− 0.13, 22.64)†				− 1.90	− 2.05 (− 6.79, 2.69)	− 2.12 (− 6.94, 2.70)
Safe to cross the road										
More dangerous to cross	152 (23.6)	− 2.65	− 10.20 (− 21.99, 1.58)†	− 9.41 (− 21.58, 2.75)†	2.63	11.05 (− 2.16, 24.26)†	14.01 (− 0.84, 28.85)†	5.52	5.88 (0.89, 10.87)⁎	5.22 (0.07, 10.37)⁎⁎
No change (reference)	324 (50.3)	4.67	0	0	− 11.37	0	0	0.75	0	0
Safer to cross	168 (26.1)	5.58	0.53 (− 11.07, 12.12)	− 0.12 (− 11.80, 11.56)	− 0.10	7.46 (− 5.26, 20.17)†	9.56 (− 4.69, 23.82)	− 2.56	− 2.56 (− 7.36, 2.23)	− 2.45 (− 7.40, 2.50)
Dangerous to cycle										
More dangerous to cycle	181 (28.3)				− 8.63	− 1.66 (− 14.99, 11.67)		7.49	7.97 (2.93, 13.01)⁎⁎	7.57 (2.40, 12.74)⁎⁎
No change (reference)	308 (48.1)		NT	NT	− 5.84	0	NS	− 0.93	0	0
Less dangerous to cycle	151 (23.6)				− 2.81	2.14 (− 10.41, 14.68)		− 1.12	− 0.46 (− 5.20, 4.29)	− 0.01 (− 4.89, 4.87)
Convenient cycle routes										
Less convenient routes	136 (21.0)				− 4.67	− 4.37 (− 17.68, 8.93)		2.25	3.54 (− 1.43, 8.52)†	2.63 (− 2.53, 7.79)
No change (reference)	380 (58.6)		NT	NT	− 8.72	0	NS	1.86	0	0
More convenient routes	133 (20.4)				1.21	3.64 (− 9.35, 16.62)		− 1.75	− 2.16 (− 7.11, 2.79)	− 1.52 (− 6.57, 3.53)

Model 1: Adjusted for age, gender, education, season of questionnaire completion and baseline travel behaviour.

Model 2: Adjusted for age, gender, education, season of questionnaire completion, baseline travel behaviour, relocation of home or work, change in workplace car parking and occurrence of any life events.

NT: Not tested. NS: Not significant and thus not carried forward to Model 2.

Route characteristics were matched to the specific behaviour of interest: walking models included pleasantness and convenience of routes for walking and convenience of public transport and cycling models included convenience of routes for cycling. Data collected in 2009 and 2010 in Cambridge, UK.

† p < 0.25.

⁎ p < 0.05.

⁎⁎ p < 0.01.

⁎⁎⁎ p < 0.001.

#### Uptake of walking, cycling and alternatives to the car

Results of these models were similar, or at least not contradictory, to those using continuous outcome measures (Table 5). Those who reported more convenient public transport (OR: 3.31, 95% CI: 1.27, 8.63) or that it was safer to cycle (OR: 3.70, 95% CI: 1.44, 9.50) over time were more likely to take up alternatives to the car.

**Table 5:** Associations between changes in route perceptions and uptake of walking, cycling and alternatives to the car.

	Took up any walking on the commute Odds ratio (95% CI)		Took up any cycling on the commute Odds ratio (95% CI)		Took up an alternative to the car on the commute Odds ratio (95% CI)	
	Model 1	Model 2	Model 1	Model 2	Model 1	Model
Pleasant to walk						
Less pleasant	0.79 (0.40, 1.56)	0.78 (0.39, 1.56)			0.97 (0.37, 2.59)	
No change (reference)	1		NT	NT	1	NS
More pleasant	1.54 (0.82, 2.89)†	1.46 (0.76, 2.80)			1.57 (0.73, 4.26)	
Convenient public transport						
Less convenient public transport	1.13 (0.62, 2.06)				0.32 (0.09, 1.18)†	0.30 (0.08, 1.18)†
No change (reference)	1	NS	NT	NT	1	1
More convenient public transport	0.94 (0.48, 1.83)				2.82 (1.13, 7.06)⁎	3.31 (1.27, 8.63)⁎
Little traffic						
More traffic	1.11 (0.60, 2.07)		1.20 (0.43, 3.33)	1.14 (0.37, 3.45)	0.76 (0.27, 2.13)	
No change (reference)	1	NS	1		1	NS
Less traffic	0.99 (0.51, 1.93)		1.72 (0.71, 4.17)†	1.78 (0.70, 4.47)†	0.91 (0.33, 2.54)	
Convenient walking routes						
Less convenient routes	0.78 (0.39, 1.55)	0.71 (0.35, 1.46)			0.57 (0.20, 1.64)	
No change (reference)	1		NT	NT		NS
More convenient routes	1.63 (0.90, 2.96)†	1.61 (0.88, 2.94)†			1.47 (0.63, 3.41)	
Safe to cross the road						
More dangerous to cross	0.68 (0.36, 1.26)†	0.62 (0.31, 1.23)†	0.88 (0.31, 2.50)	0.84 (0.27, 2.61)	0.64 (0.20, 2.09)	0.61 (0.18, 2.04)
No change (reference)	1		1		1	
Safer to cross	0.65 (0.34, 1.26)†	0.58 (0.29, 1.16)†	1.99 (0.87, 4.56)†	1.76 (0.75, 4.17)†	1.88 (0.80, 4.43)†	2.15 (0.86, 5.36)†
Dangerous to cycle						
More dangerous to cycle			0.92 (0.33, 2.55)	0.83 (0.27, 2.50)	0.24 (0.05, 1.13)†	0.23 (0.05, 1.15)†
No change (reference)	NT	NT	1		1	
Less dangerous to cycle			1.88 (0.82, 4.30)†	1.69 (0.71, 4.06)†	3.57 (1.42, 8.96)⁎	3.70 (1.44, 9.50)⁎⁎⁎
Convenient cycle routes						
Less convenient routes			0.78 (0.29, 2.12)		0.65 (0.25, 1.71)⁎⁎	
No change (reference)	NT	NT	1	NS	1	NS
More convenient routes			1.37 (0.60, 3.11)		1.09 (0.44, 2.64)	

Model 1: Adjusted for age, gender, education and season of questionnaire completion.

Model 2: Adjusted for age, gender, education, season of questionnaire completion, relocation of home or work, change in workplace car parking and occurrence of any life events.

NT: Not tested. NS: Not significant and thus not carried forward to Model 2.

Data collected in 2009 and 2010 in Cambridge, UK. Route characteristics were matched to the specific behaviour of interest: walking models included pleasantness and convenience of routes for walking and convenience of public transport and cycling models included convenience of routes for cycling.

† p < 0.25.

⁎ p < 0.05.

⁎⁎ p < 0.01.

⁎⁎⁎ p < 0.001.

## Discussion

### Principal findings

Commuters who reported that routes had become less pleasant for walking or more dangerous for cycling, or that roads had become more difficult to cross, were more likely to report an increase in car trips, a decrease in time spent walking or both. Increases in perceived convenience of public transport and safety for cycling were associated with uptake of alternatives to the car.

### Consistency of results across outcomes and environmental changes

The findings from the analyses of uptake, and of changes in weekly duration of walking and cycling, were complementary but not identical. The analyses of uptake compared participants who took up any walking or cycling with those who never reported the behaviours and were therefore restricted to a subsample of participants, whereas continuous measures of changes in time spent walking and cycling were computed for all participants. Whilst those who reported less supportive conditions for walking and cycling over time reported an increase in car trips and (to a lesser extent) a decrease in time spent walking, these associations were not mirrored by significant changes in the opposite direction associated with positive environmental changes. However, the *directions* of the effects were consistent in that the point estimates of the regression coefficients associated with positive and negative environmental exposures were generally of opposite signs. Consistent with the observation that environmental changes may be ‘necessary but not sufficient’ to promote physical activity (Giles-Corti and Donovan, 2002), it may be necessary to address both the barriers to and facilitators of physical activity behaviours to achieve sustained behaviour change. However, the lack of consistent statistical significance across all analyses highlights the need for rigorous evaluation to confirm the effects of environmental interventions in practice.

### Active travel and car use

The associations observed between changes in environmental perceptions and changes in car use were not simply the inverse of the associations with active travel. This may be partly explained by the fact that these behaviours are not mutually exclusive: in this study, 31% of car users reported some walking and cycling in combination with car use at t_1_ (Panter et al., 2013b). The different patterns of associations suggest that some environmental interventions (e.g. improving pedestrian routes) may be more effective in promoting walking and cycling without necessarily reducing car trips, whereas others (e.g. changes in parking provision) may be more effective in reducing car trips.

### Potential targets for intervention and avenues for future research

Changes in only a few specific perceptions of the route environment were associated with changes in commuting behaviour. Together with our previous paper (Panter et al., 2013a), our complementary approaches to longitudinal analysis strengthen the evidence for causality (Bauman et al., 2002) and the case for the evaluation of interventions aiming to provide safe, convenient routes for walking and cycling and convenient public transport. These findings are consistent with the conclusion of a recent systematic review that studies with designs capable of supporting more robust causal inference in this field (e.g. those attempting to assess temporal precedence) tend to find more null associations than cross-sectional studies (McCormack and Shiell, 2011).

In keeping with previous research (Humpel et al., 2002, Humpel et al., 2004), we found that those who reported unsupportive conditions for walking or cycling at t_1_ tended to report that conditions had improved at t_2_, whilst those who already perceived the environment to be supportive tended to report no change or small decreases. This may represent regression to the mean (Barnett et al., 2005). Further research using multiple measures over time may help to disentangle effects of regression to the mean on exposure or outcome measurement in cohorts. Quasi-experimental studies that specify and test casual pathways leading to behaviour change would also provide more rigorous assessment of the effects of environmental change on walking and cycling (Bauman et al., 2002).

Researchers studying changes in travel behaviour have used a variety of metrics including changes in trip frequency (Hume et al., 2009) or in time spent walking or cycling (Humpel et al., 2004) or uptake of specific behaviours (Beenackers et al., 2012, Cleland et al., 2008, Sugiyama et al., 2013), all of which relate to different research questions. Changes in reported time spent walking or cycling can be used to infer changes in time spent in moderate-to-vigorous intensity physical activity and consequent quantifiable health benefits, but such changes may largely reflect existing walkers or cyclists making more or longer trips (Ogilvie et al., 2004) or self-report measurement error (Rissel et al., 2010). Measures of uptake of new behaviours, including switching between usual modes of travel, may therefore also be valuable, particularly for understanding the effectiveness of interventions in promoting activity among the less active. In summary, analysis of multiple outcome measures in combination may help to ensure that robust conclusions are drawn.

### Strengths and limitations

Key strengths of this study include the large longitudinal sample of urban and rural working adults and the use of several complementary metrics of travel behaviour change. Given that changes in environmental conditions and commuting were assessed concurrently, we cannot tell from this analysis alone whether changes in perceptions resulted in changes in behaviour or vice versa. Although it is possible that behaviour change may have resulted in altered environmental perceptions, such behaviour change would likely have been prompted by other factors. Our results were unchanged after adjustment for other factors shown to influence commuting decisions (Jones and Ogilvie, 2012, Scheiner and Holz-Rau, 2013) and largely consistent with those of our analysis of baseline predictors of change (Panter et al., 2013a), suggesting that it is more likely that the changes in environmental perceptions preceded the behaviour changes.

The high prevalence of walking and cycling in this sample allowed us to examine a suite of complementary metrics of changes in outcomes, but our findings may not be generalisable to other contexts, particularly those where cycling is less prevalent. Our sample was relatively affluent and well educated and only 56% of initial participants provided data at follow-up. Although baseline travel behaviour was not associated with dropout, the composition and attrition of the cohort somewhat limits the generalisability of our results. Women are overrepresented in the sample and this may have limited the precision of our estimates for men. Our outcome measures were based on changes in past-week commuting at each time point, and may therefore have been subject to short term fluctuations rather than representing longer term patterns. We also cannot exclude the possibility of wider influences on behaviour change, such as changes in fuel prices or public transport fares.

## Conclusions

Taken together with previous research, these findings confirm the potential role of environmental interventions to promote walking and cycling, particularly those addressing the safety and pleasantness of walking and cycling routes and the convenience of public transport. These should be rigorously evaluated.

## Conflict of interest

The authors declare that there is no conflict of interest.
