# Supplementary material for: Active commuting and perceptions of the route environment: A longitudinal analysis
Source: Prev Med. 2014 Oct;67:134–40. doi: 10.1016/j.ypmed.2014.06.033 (PMC4175182; doi:10.1016/j.ypmed.2014.06.033)
Supplement: Supplementary file 1 — Supplementary material. [file mmc1.doc]

**Additional file A:** Details of secondary outcome measures used

| Outcome | Variable  used to define change | Predictor group | | Reference group | | Sample size used in analysis** |
| --- | --- | --- | --- | --- | --- | --- |
|  |  | Description | Sample size* | Description | Sample size* |  |
| Uptake of walking | Weekly time spent walking | Increased walking (from 0 at t_1_ to >0 at t_2_) (‘*took up walking’)* | 72 | Spent no time walking at either time point (‘*no walking’)* | 401 | 470 |
| Uptake of cycling | Weekly time spent cycling | Increased cycling (from 0 at t_1_ to >0 at t_2_) (‘*took up cycling’)* | 33 | Spent no time cycling at either time point (‘*no cycling’)* | 268 | 293 |
| Uptake of alternatives to the car | Most frequently reported mode(s) | Shifted from car to alternative usual mode | 37 | Car user at both time points | 137 | 174 |

*Sample size refers to potential numbers of participants in each group (not accounting for missing data in potential predictors)

**Sample size refers to actual number of participants used in maximally adjusted models (those with complete data for all predictors included in the model).

Data collected in 2009 and 2010 in Cambridge, UK

**Additional file B:** Time spent walking and cycling and car trips at baseline and follow-up stratified by age and gender

|  | Median (IQR) | | | | | | |
| --- | --- | --- | --- | --- | --- | --- | --- |
|  | Weekly time spent walking (min) | | Weekly time spent cycling (min) | | Percentage of  car-only trips | | |
|  | Baseline | Follow-up | Baseline | Follow-up | | Baseline | Follow-up |
| All sample | 0 (0, 25) | 0 (0, 35) | 50 (0, 150) | 0 (40, 150) | | 0 (0, 50) | 0 (0, 60) |
| Gender |  |  |  |  | |  |  |
| Men | 0 (0, 0) | 0 (0, 20) | 100 (0, 180) | 80 (0, 150) | | 0 (0, 25) | 0 (0, 40) |
| Women | 0 (0, 40) | 0 (0, 40) | 0 (0, 150) | 0 (0, 140) | | 0 (0, 59) | 0 (0, 73) |
| Age group |  |  |  |  | |  |  |
| <30 years | 0 (0, 40) | 0 (0, 45) | 80 (0, 156) | 60 (0, 150) | | 0 (0, 14) | 0 (0, 10) |
| 30-50 years | 0 (0, 20) | 0 (0, 40) | 40 (0, 160) | 40 (0, 150) | | 0 (0, 50) | 0 (0, 60) |
| 50+ years | 0 (0, 25) | 0 (0, 20) | 60 (0, 150) | 50 (0, 150) | | 0 (0, 50) | 0 (0, 60) |

**Additional file C:** Maximally adjusted associations between magnitude of changes in environmental perceptions and changes in time spent walking or cycling and in proportion of car trips

|  |  | Change in time spent walking  on the commute (mins/week) | | Change in time spent cycling  on the commute (mins/week) | | Change in percentage of car-only  trips | |
| --- | --- | --- | --- | --- | --- | --- | --- |
|  | **N** | Minimal adjustment | Maximal adjustment | Minimal adjustment | Maximal adjustment | Minimal adjustment | Maximal adjustment |
| **Pleasant to walk** |  |  |  |  |  |  |  |
| Decrease of >1 point | 7.5 (47) | -13.40 (-32.76, 5.96) | -13.33 (-32.30, 5.65) |  |  | 8.24 (0.15, 16.34) | 7.68 (-0.44, 15.81) |
| Decrease of 1 point | 21.4 (134) | -11.30 (-24.27, 1.66) | -11.75 (-24.55, 1.06) |  |  | 5.26 (-0.21, 10.73) | 5.80 (0.27, 11.32) |
| No change (reference) | 49.9 (312) | 0* | 0† | NT | NT | 0† | 0† |
| Increase of 1 point | 14.7 (92) | -7.68 (-22.51, 7.15) | -7.89 (-22.43, 6.64) |  |  | 4.91 (-1.28, 11.11) | 3.26 (-2.94, 9.47) |
| Increase of >1 point | 6.4 (40) | 15.98 (-5.17, 37.13) | 15.63 (-4.87, 36.12) |  |  | -0.34 (-9.19, 8.51) | -1.05 (-9.82, 7.73) |
| **Convenient public transport** |  |  |  |  |  |  |  |
| Decrease of >1 point | 8.7 (56) | 1.85 (-16.10, 19.81) | 1.64 (-15.90, 19.18) |  |  | 2.03 (-5.50, 9.56) | 1.51 (-5.99, 9.01) |
| Decrease of 1 point | 17.5 (112) | 3.41 (-10.29, 17.11) | 3.89 (-9.71, 17.49) |  |  | 0.51 (-5.23, 6.24) | -0.10 (-5.91, 5.71) |
| No change (reference) | 53.1 (340) | 0† | 0 | NT | NT | 0† | 0 |
| Increase of 1 point | 14.2 (91) | 9.64 (-4.96, 24.25) | 8.11 (-6.47, 22.69) |  |  | -4.66 (-10.76, 1.45) | -4.73 (-10.95, 1.49) |
| Increase of >1 point | 6.4 (41) | 21.90 (1.38, 42.42) | 15.08 (-5.16, 35.32) |  |  | -1.45 (-10.01, 7.10) | -1.90 (-10.49, 6.70) |
| **Little traffic** |  |  |  |  |  |  |  |
| Decrease of >1 point | 5.4 (35) | -2.56 (-25.00, 19.88) | -13.21 (-36.35, 9.93) | 9.54 (-14.83, 33.91) | 11.44 (-14.26, 37.14) | -0.08 (-9.47, 9.32) | 0.62 (-9.27, 10.50) |
| Decrease of 1 point | 15.5 (101) | 0.67 (-13.39, 14.73) | 1.29 (-12.52, 15.11) | 12.25 (-3.05, 27.54) | 11.23 (-4.15, 26.62) | -1.03 (-6.91, 4.86) | -0.41 (-6.33, 5.50) |
| No change (reference) | 58.6 (380) | 0 | 0 | 0 | 0 | 0 | 0 |
| Increase of 1 point | 14.9 (97) | -5.87 (-20.05, 8.31) | -6.60 (-20.53, 7.34) | 2.92 (-12.50, 18.35) | 2.60 (-12.92, 18.11) | 0.71 (-5.22, 6.64) | 0.88 (-5.08, 6.84) |
| Increase of >1 point | 5.5 (36) | 8.78 (-12.72, 30.28) | 5.13 (-17.01, 27.27) | -3.89 (-27.22, 19.44) | -0.63 (-25.26, 23.99) | -0.68 (-9.70, 8.35) | 0.06 (-9.44, 9.57) |
| **Convenient walking routes** |  |  |  |  |  |  |  |
| Decrease of >1 point | 8.8 (57) | 9.43 (-8.45, 27.30) | 9.75 (-7.92, 27.42) |  |  | 4.84 (-2.51, 12.20) | 1.78 (-5.66, 9.21) |
| Decrease of 1 point | 16.5 (106) | 9.52 (-4.63, 23.68) | 5.26 (-8.74, 19.26) |  |  | 2.81 (-3.01, 8.63) | 3.26 (-2.62, 9.14) |
| No change (reference) | 45.9 (295) | 0 | 0 | NT | NT | 0† | 0 |
| Increase of 1 point | 18.7 (120) | 7.35 (-6.05, 20.75) | 8.05 (-5.09, 21.20) |  |  | -3.82 (-9.33, 1.70) | -4.16 (-9.69, 1.37) |
| Increase of >1 point | 9.97 (64) | 17.27 (0.18, 34.37) | 17.52 (0.88, 34.16) |  |  | 1.83 (-5.22, 8.89) | 1.78 (-5.23, 8.79) |
| **Safe to cross the road** |  |  |  |  |  |  |  |
| Decrease of >1 point | 7.5 (48) | 9.43 (-8.45, 27.30) | -11.49 (-30.50, 7.51) | 11.34 (-9.54, 32.23) | 12.57 (-8.55, 33.69) | 10.54 (2.65, 18.43) | 9.22 (1.25, 17.19) |
| Decrease of 1 point | 16.1 (104) | 9.52 (-4.63, 23.68) | -8.53 (-22.45, 5.39) | 12.94 (-2.41, 28.28) | 10.58 (-4.91, 26.06) | 2.96 (-2.84, 8.76) | 3.49 (-2.36, 9.33) |
| No change (reference) | 50.3 (324) | 0 | 0 | 0 | 0 | 0* | 0* |
| Increase in 1 point | 18.5 (119) | 7.35 (-6.05, 20.75) | -3.44 (-16.61, 9.73) | 6.00 (-8.47, 20.46) | 4.41 (-10.25, 19.06) | -0.67 (-6.13, 4.79) | 0.03 (-5.49, 5.55) |
| Increase of >1 point | 7.6 (49) | 17.27 (0.18, 34.37) | 7.11 (-12.02, 26.24) | 10.59 (-10.28, 31.46) | 9.40 (-11.96, 30.76) | -7.41 (-15.30, 0.48) | -8.03 (-16.09, 0.03) |
| **Dangerous to cycle** |  |  |  |  |  |  |  |
| Decrease of >1 point | 7.0 (45) |  |  | -2.17 (-23.62, 19.28) | -6.30 (-28.11, 15.52) | 13.28 (5.15, 21.41) | 13.55 (5.27, 21.82) |
| Decrease in 1 point score | 16.6 (106) |  |  | 1.91 (-13.41, 17.22) | 1.93 (-13.37, 17.24) | 5.56 (-0.24, 11.36) | 5.18 (-0.63, 10.98) |
| No change (reference) | 48.1 (308) | NT | NT | 0 | 0 | 0*** | 0*** |
| Increase of 1 point | 20.6 (132) |  |  | 4.81 (-9.26, 18.88) | 3.18 (-11.00, 17.36) | -1.42 (-6.75, 3.91) | -1.13 (-6.51, 4.24) |
| Increase of >1 point | 7.6 (49) |  |  | -1.25 (-22.19, 19.68) | -2.71 (-23.83, 18.42) | 1.61 (-6.32, 9.55) | 1.63 (-6.39, 9.64) |
| **Convenient cycle routes** |  |  |  |  |  |  |  |
| Decrease of >1 point | 7.7 (50) |  |  | -17.15 (-37.62, 3.32) | -14.32 (-35.05, 6.41) | 4.98 (-2.82, 12.77) | 3.15 (-4.74, 11.03) |
| Decrease of 1 point | 15.0 (97) | NT | NT | 1.03 (-14.75, 16.82) | 1.93 (-13.91, 17.77) | 1.22 (-4.79, 7.23) | 1.66 (-4.36, 7.68) |
| No change (reference) | 53.0 (342) |  |  | 0 | 0 | 0 | 0 |
| Increase in 1 point | 16.6 (107) |  |  | 3.79 (-11.29, 18.87) | 5.24 (-9.95, 20.42) | -1.94 (-7.67, 3.80) | -1.58 (-7.35, 4.18) |
| Increase of >1 point | 7.6 (49) |  |  | 1.44 (-19.09, 21.97) | 1.55 (-19.28, 22.39) | -1.18 (-9.01, 6.64) | -1.56 (-9.50, 6.37) |

† p<0.25; * p<0.05; **p<0.01; *** p<0.001*Sample size refers to potential numbers of participants in each group (not accounting for missing data in potential predictors)**Sample size refers to actual number of participants used in maximally adjusted models (those with complete data for all predictors included in the model).

Route characteristics were matched to the specific behaviour of interest: walking models included pleasantness and convenience of routes for walking and convenience of public transport and cycling models included convenience of routes for cycling. Data collected in 2009 and 2010 in Cambridge, UK
